# Shoulder arthroplasty in the osteoporotic patient: do bisphosphonates make a difference?

**DOI:** 10.1016/j.jseint.2026.101629

**Published:** 2026-01-28

**Authors:** Nathaniel C. Adams, Nicholas Bank, Chandler Q. Kotseos, Jonathan R. Davis, Jack R. Felkner, Robert A. Creighton, Ganesh M. Kamath

**Affiliations:** a Department of Orthopaedics, The University of North Carolina, Chapel Hill, NC, USA; b The University of North Carolina School of Medicine, Chapel Hill, NC, USA; c Department of Orthopaedics, Case Western Reserve University School of Medicine, Cleveland, OH, USA

**Keywords:** Osteoporosis, Shoulder arthroplasty, Surgical outcomes, Bisphosphonates, Epidemiology, PJI

## Abstract

**Background:**

Osteoporosis (OP) affects much of the global population with a prevalence of 23.1% in women and 11.7% in men. This is relevant to orthopedists as osteoporotic patients have more postoperative complications following arthroplasty than nonosteoporotic patients. OP is also a risk factor for poorer medical and implant-related complications postoperatively for total shoulder arthroplasty (TSA) patients. To improve bone quality in this population, bisphosphonates (BP) are routinely utilized. This study aims to determine any differences in postoperative outcomes for osteoporotic TSA patients who are and aren't medically managed for their OP.

**Methods:**

The TriNetX database was queried to identify BP-managed or unmanaged osteoporotic TSA patients. Variables were identified using diagnosis and procedural codes. Patients were included if they had OP and underwent primary TSA and excluded if they had prior shoulder hemiarthroplasty, prior prescription for OP drugs other than BPs, or lacked a minimum of 2 years follow-up. Patients were propensity-matched into 2 cohorts: (1) those receiving BP therapy within 3 years of the index surgery (BP group) and (2) patients without any history of OP treatment (noTx group). Rates of periprosthetic fracture, postoperative infection, prosthetic joint infection (PJI), intraoperative fracture, osteolysis, mechanical loosening, dislocation, and revision surgery were examined at 3 months, 1 year, and 2 years postoperatively. Outcomes between cohorts were compared using odds ratios. All statistical analyses were performed via TriNetX in-suite software.

**Results:**

We identified 7,208 patients between the BP and noTx groups. Average ages for the BP group and noTx group were 73.6 ± 8.6 and 73.6 ± 9 years respectively. The BP group was less likely to experience periprosthetic fracture [0.7% vs. 1.2%, odds ratio (OR) 0.565 (0.345, 0.925)], PJI [0.8% vs. 1.8%, OR 0.45 (0.292, 0.695)], and revision TSA [0.8% vs. 1.4%, OR 0.59 (0.371, 0.934)] within 90 days, PJI [1.6% vs. 2.7%, OR 0.56 (0.401, 0.778)], revision TSA [1.6% vs. 2.6%, OR 0.62 (0.443, 0.854)], and mechanical loosening [0.8% vs. 1.4%, OR 0.57 (0.365, 0.901)] at 1 year, and PJI [2.1% vs. 3.4%, OR 0.61 (0.459, 0.817)] and mechanical loosening [1.4% vs. 2.1%, OR 0.67 (0.466, 0.953)] at 2 years. There were no significant differences in rates of non-PJI postoperative infection, or dislocation at 90 days, 1 year, or 2 years.

**Conclusion:**

Bisphosphonate-treated OP was associated with lower rates of periprosthetic fracture, osteolysis, and revision TSA within 90 days, revision TSA at 1 year, and PJI and mechanical loosening at both 1 and 2 years.

Osteoporosis (OP) affects a significant portion of the population with an incidence of 23.1% in women and 11.7% in men globally.^27^ Historically, patients with OP who undergo joint replacement have consistently poorer outcomes including malpositioning, implant loosening, and subsidence when compared to nonosteoporotic patients.^3^^,^^5^^,^^8^^,^^17^ More recently, OP was found to be an independent risk factor for poorer medical and implant-related complications following total shoulder arthroplasty (TSA).^16^ Considering these complications and that upwards of 26.2% of TSA patients have OP, it is increasingly important to understand the factors affecting patient outcomes in this population.^5^

Many OP medications, most commonly bisphosphonates (BP), are routinely utilized in efforts to improve bone quality in these patients.^25^^,^^29^^,^^31^ While reports in osteoporotic patients undergoing arthroplasty can be favorable, there are still disparities regarding surgical outcomes associated with perioperative BP use. For example, benefits of these medications have been demonstrated in the surgical management for hip fractures, including arthroplasty, where BP-managed osteoporotic patients had improved functional outcomes than those without BPs.^20^ Conversely, BPs taken prior to TSA were associated with increased rates of intraoperative fracture and more postoperative complications at 1 year, but no statistically significant differences at 2 years compared to controls.^19^ Given such discrepancies, the aim of this study is to determine the effects of perioperative BP use on postsurgical outcomes following TSA in osteoporotic patients.

## Methods

### Study design, data collection, and study population

Data for this retrospective cohort study were obtained using TriNetX, a global federated health research network which enables querying of deidentified health records for demographic information, diagnoses, procedures, medications, and laboratory values using standardized coding systems such as International Classification of Diseases (ICD), Current Procedural Terminology (CPT) and RxNorm. The TriNetX Research Network (comprising 109 health-care organizations and nearly 150 million patient records) was queried to identify patients with OP who underwent TSA. As data in TriNetX are deidentified per the deidentification standard defined in Section §164.514(a) of The Health Insurance Portability and Accountability Act of 1996 (HIPAA) Privacy Rule and attested to through a formal determination by a qualified expert as defined in Section §164.514(b) (1) of the HIPAA Privacy Rule (refreshed in December 2020), this study is exempt from informed consent and institutional review board approval. This study adhered to the Strengthening the Reporting of Observational Studies in Epidemiology guidelines for consistent reporting of observational data.^7^

### Cohort selection and propensity matching

Patients, procedures and postoperative complications were identified using ICD-10 and CPT codes. Patients were included if they underwent either anatomic or reverse TSA and a diagnosis of OP within 3 months of TSA. Patients were excluded if they had a history shoulder hemiarthroplasty or prescription for a parathyroid hormone analog or monoclonal antibody treatment for OP. Patients without a minimum of 2 years follow-up were also excluded. Patients meeting inclusion and exclusion criteria were stratified into 2 cohorts: (1) those receiving BP therapy within 3 years of the index surgery (BP group) and (2) patients without any OP treatment (noTx group). Cohorts were matched 1:1 via propensity score matching (PSM) based on age at index event, sex, race, and the Charlson Comorbidity Index. Significance was held at *P* < .007 after Bonferroni correction for multiple comparisons between the cohorts. ICD-10 and CPT codes used for patient identification and PSM can be found in the Supplementary File.

### Outcomes

The primary outcomes of interest were rates of periprosthetic fracture, postoperative infection, prosthetic joint infection (PJI), intraoperative fracture, osteolysis, mechanical loosening, dislocation, and revision surgery were examined at 3 months, 1 and 2 years postoperatively. ICD-10 and CPT codes for identifying outcomes of interest can be found in Supplementary File.

### Statistical analysis

Chi-square tests were used to determine differences in categorical demographic and comorbidity variables, and Student's t-tests were used to analyze differences in continuous variables. Outcomes between cohorts were compared using odds ratios (ORs) with 95% confidence intervals. A *P* value of <.05 was used to determine statistical significance for surgical outcomes. All statistical analyses were performed using the TriNetX in-suite analytic software.

## Results

### Demographics

We identified 3604 BP-managed osteoporotic TSA patients and 3,604 non–medically managed osteoporotic TSA patients after PSM. The average ages for the BP group and noTx group were 73.6 ± 8.6 years and 73.6 ± 9 years, respectively (*P* = .877). Sex in terms of percentage of females (86.4% vs. 86.8%, *P* = .653) and race in terms of percent White (84.1% vs. 84.9%, *P* = .313) were also very similar between groups. All patients in the BP group had a bisphosphonate prescription within 3 years preoperatively and two-third of patients still had a prescription for a bisphosphonate at 2 years postoperatively. Further demographic data are displayed in Table I with outcomes results summarized in Table II, Table III, Table IV.

**Table I:** Cohort demographics of BP and noTx groups before and after propensity matching.

Demographics	Unmatched cohort			Matched cohort		
	BP group N = 3,642	noTx group N = 6,775	*P* value	BP group N = 3,604	noTx group N = 3,604	*P* value
Age (yr ± SD)	73.6 ± 8.6	73.3 ± 9.4	.074	73.6 ± 8.6	73.6 ± 9.0	.877
Male (%)	9.50%	17.30%	<.001	9.6%	9.6%	1.000
Female (%)	86.5%	76.3%	<.001	86.4%	86.8%	.653
White (%)	84.2%	80.8%	<.001	84.1%	84.9%	.313
Black (%)	5.6%	5.9%	.572	5.6%	5.4%	.718
Asian (%)	1.2%	1.3%	.562	1.2%	0.8%	.102
BMI ± SD	28.5 ± 6.7	29.2 ± 7.0	<.001	28.5 ± 6.7	28.7 ± 6.8	.435

*BP group*, bisphosphonate-treated group; *noTx group*, no treatment group; *BMI*, body mass index; *N*, cohort size; *SD*, standard deviation.

Percentages are a proportion of each cohort with given variable. Means are expressed with standard deviation.

**Table II:** Ninety-day odds ratios and 95% confidence intervals for proportion of patients with postoperative complications in bisphosphonate-managed OP patients compared to nonmedically managed OP patients.

Complication	BP group N = 3,604		noTx group N = 3,604		OR	95% CI	*P* value
	n	%	n	%			
Dislocation	36	1.0%	44	1.2%	0.82	(0.524, 1.271)	.368
Intraoperative fracture	10	0.3%	10	0.3%	1.00	(0.416, 2.405)	1.000
Mechanical loosening	13	0.4%	25	0.7%	0.52	(0.265, 1.015)	.051
Osteolysis	10	0.3%	10	0.3%	1.00	(0.416, 2.405)	1.000
Periprosthetic fracture	25	0.7%	44	1.2%	0.57	**(0.345, 0.925)**	**.022**
Postoperative infection	28	0.8%	42	1.2%	0.66	(0.411, 1.074)	.093
Prosthetic joint infection	30	0.8%	66	1.8%	0.45	**(0.292, 0.695)**	**.000**
Revision surgery	29	0.8%	49	1.4%	0.59	**(0.371, 0.934)**	**.023**

*BP group*, bisphosphonate-treated group; *noTx group*, no treatment group; *OR*, odds ratio; *CI*, confidence interval; *OP*, osteoporosis.

Bold signifies statistical significance (*P* ≤ .05).

**Table III:** One-year odds ratios and 95% confidence intervals for proportion of patients with postoperative complications in bisphosphonate-managed OP patients compared to nonmedically managed OP patients.

Complication	BP group N = 3,604		noTx group N = 3,604		OR	95% CI	*P* value
	n	%	n	%			
Dislocation	65	1.8%	68	1.9%	0.96	(0.678, 1.346)	.793
Intraoperative fracture	10	0.3%	10	0.3%	1.00	(0.416, 2.405)	1.000
Mechanical loosening	30	0.8%	52	1.4%	0.57	**(0.365, 0.901)**	**.015**
Osteolysis	10	0.3%	10	0.3%	1.00	(0.416, 2.405)	1.000
Periprosthetic fracture	45	1.2%	65	1.8%	0.69	(0.469, 1.010)	.055
Postoperative infection	46	1.3%	65	1.8%	0.70	(0.481, 1.030)	.069
Prosthetic joint infection	56	1.6%	99	2.7%	0.56	**(0.401, 0.778)**	**.000**
Revision surgery	59	1.6%	95	2.6%	0.62	**(0.443, 0.854)**	**.003**

*BP group*, bisphosphonate-treated group; *noTx group*, no treatment group; *OR*, odds ratio; *CI*, confidence interval; *OP*, osteoporosis.

Bold signifies statistical significance (*P* ≤ .05).

**Table IV:** Two-year odds ratios and 95% confidence intervals for proportion of patients with postoperative complications in bisphosphonate-managed OP patients compared to nonmedically managed OP patients.

Complication	BP group N = 3,604		noTx group N = 3,604		OR	95% CI	*P* value
	n	%	n	%			
Dislocation	94	2.6%	82	2.3%	1.15	(0.852, 1.552)	.360
Intraoperative fracture	10	0.3%	10	0.3%	1.00	(0.416, 2.405)	1.000
Mechanical loosening	51	1.4%	76	2.1%	0.67	**(0.466, 0.953)**	**.025**
Osteolysis	10	0.3%	10	0.3%	1.00	(0.416, 2.405)	1.000
Periprosthetic fracture	62	1.7%	79	2.2%	0.78	(0.558, 1.093)	.148
Postoperative infection	72	2.0%	88	2.4%	0.81	(0.594, 1.116)	.201
Prosthetic joint infection	77	2.1%	124	3.4%	0.61	**(0.459, 0.817)**	**.001**
Revision surgery	93	2.6%	116	3.2%	0.80	(0.604, 1.050)	.106

*BP group*, bisphosphonate-treated group; *noTx group*, no treatment group; *OR*, odds ratio; *CI*, confidence interval; *OP*, osteoporosis.

Bold signifies statistical significance (*P* ≤ .05).

### Surgical outcomes

The BP group were statistically less likely than the noTx group to have had periprosthetic fracture [0.7% vs. 1.2%, OR 0.565 (0.345, 0.925)], PJI [0.8% vs. 1.8%, OR 0.45 (0.292, 0.695)], and revision TSA [0.8% vs. 1.4%, OR 0.59 (0.371, 0.934)] within 90 days of surgery. At 1-year postoperatively, the BP group was also less likely than the noTx group to experience PJI [1.6% vs. 2.7%, OR 0.56 (0.401, 0.778)], revision TSA [1.6% vs. 2.6%, OR 0.62 (0.443, 0.854)], and mechanical loosening [0.8% vs. 1.4%, OR 0.57 (0.365, 0.901)]. Additionally, the BP group had a significantly lower likelihood than the noTx group for PJI [2.1% vs. 3.4%, OR 0.61 (0.459, 0.817)] and mechanical loosening [1.4% vs. 2.1%, OR 0.67 (0.466, 0.953)] at 2 years. There were no significant differences in rates of dislocation, intraoperative fracture, osteolysis, and non-PJI postoperative infection within 90 days, 1 year, or 2 years after surgery between the BP and noTx groups. Notably, rates of PJI were significantly lower in the BP group when compared to the noTx over all measured time periods.

## Discussion

The most significant finding of this study is that BP-managed osteoporotic patients undergoing primary TSA demonstrated lower rates of postoperative implant-related complications (periprosthetic fracture, prosthetic joint infection, and revision TSA within 90 days, revision TSA at 1 year, and PJI and mechanical loosening at both 1 and 2 years) compared to osteoporotic patients without any BPs. The strength of reduction in odds within 90 days for development of PJI was strong and the strength of reduction for periprosthetic fracture and revision surgery were both moderate. At 1 and 2 years, the strength of odds reduction was moderate for mechanical loosening, PJI, and revision TSA. Rates of these complications in BP-managed patients were lower than nonmedically managed OP but still on average higher than historical healthy controls.^16^

There is mixed evidence surrounding the efficacy of BP treatment on arthroplasty outcomes. Certain studies looking at BP usage prior to lower extremity arthroplasty have shown no benefit or even increased rates of aseptic loosening, dislocation, periprosthetic osteolysis, and stress fracture compared to those without preoperative BP management.^13^^,^^17^ Furthermore, a meta-analysis by Lin et al looking at hip and knee arthroplasty patients found that BP-managed patients had significantly higher rates of postoperative complications with preoperative BP use but significantly lower rates of all-cause revision when BP therapy was initiated postoperatively.^18^ In accordance with these findings, one explanation for our results could be a larger proportion of patients on postoperative BP therapy as opposed to preoperative usage.

Nonetheless, arthroplasty outcomes between weight-bearing and non–weight-bearing joints may differ. In recent work looking at TSA in osteoporotic patients, we note consistency among our rates of postoperative complications.^5^^,^^16^ For example, rates of revision surgery for our noTx OP cohort at 2 years were similar to current studies' undifferentiated OP cohorts which ranged from 2.8-3.2%.^5^^,^^16^ Additionally, our rates of dislocation, periprosthetic fracture, and mechanical loosening were also consistent with prior literature.^5^^,^^16^ Interestingly, our study also identified BP-managed OP was associated with reduced postoperative prosthetic-related complications which is in opposition to findings by Mai et al. In their cohort of 87 patients (29 BP+ and 58 BP-), they identified that patients treated preoperatively with BPs demonstrated higher rates of intraoperative fracture and periprosthetic fracture at 1 year. Our cohorts did not exhibit any significant differences in intraoperative fracture at any time point, and our BP cohort had significantly lower rates of periprosthetic fracture at 90 days and lower, though not significant, rates at 1 and 2 years. Of note, Mai et al's study faced limitations with OP diagnosis heterogeneity (90% in BP + group and 40% in BP- group vs. 100% of patients in both of our cohorts), self-reported power limits, and uncertainty regarding postoperative BP usage.^19^ Accordingly, the divergence of our results may stem from larger and more homogenous cohorts.

Considering that OP leads to poorer outcomes following arthroplasty,^11^^,^^12^^,^^14^ it is worthwhile to optimize the quality of bone perioperatively.^21^^,^^32^ BPs are routinely recommended in postmenopausal women at high risk for fracture,^31^ and they improve bone mineral density by decreasing bone resorption through the inhibition of osteoclasts. This is evidenced by improved dual-energy x-ray absorptiometry scores with BP use.^1^^,^^26^ Further, these medications are quite safe with the severe complications of atypical femoral fractures and osteonecrosis of the jaw happening in less than 0.13% and 0.3% of patients respectively.^22^^,^^24^ However, even with clear guidelines for OP management, only around a third to a half of qualifying patients end up receiving any kind of medical management^10^^,^^28^; One study found nearly a third of patients undergoing TSA met criteria for OP medications, but only 20% of those that qualified received therapy.^4^ With both the incidence of OP and joint arthroplasty independently on the rise, it reasons that the intersection of these 2 groups means more osteoporotic joint replacement candidates will be receiving arthroplasties.^15^^,^^23^^,^^30^^,^^33^ In turn, appropriate treatment regimens can potentially improve outcomes for the osteoporotic arthroplasty patient. A recent meta-analysis found that an average of 12.4 months of BP therapy was required to reduce fracture risk in postmenopausal women with an average increase in T-score of 0.15-0.3 over a 12- to 24-month time period.^2^^,^^6^^,^^9^ Notably, the risk of complications from arthroplasty increases at a T-score of ≤ −2.5.^21^ Thus, by increasing bone mineral density and T-scores above this threshold, risks of postoperative implant-related complications can theoretically be decreased.^11^ In line with this, our results support the perioperative usage of BPs when medically indicated and may help guide counseling of osteoporotic patients regarding their elevated risks following TSA in addition to mitigation strategies.

### Limitations

This study, while useful to aggregate large volumes of patient data to explore relatively rare outcomes, is not without limitations. One primary limitation is the reliance on coding data for cohort and outcomes identification, which is dependent on accuracy and consistency of code entry into patient records. In efforts to mitigate this, TriNetX employs a robust data quality framework, including data harmonization, a growing data quality library, and site benchmarking. Additionally, while ICD/CPT codes allow for specifying diagnoses and procedures, identifying and controlling for nuance among variables (anatomic vs. reverse TSA, postoperative care, differences in practice standards) surrounding the index surgery is not possible with this study design and would require prospective data collection or individualized chart review. Similarly, coding data alone cannot delineate OP severity, including T- or Z-score. Further limitation lies in the fact that prescription status and medication adherence are not inextricably linked; while BP prescriptions within our specified time window are verifiable, there may be variations in dosage and frequency between patients. It is also not possible to ascertain patient adherence to their prescribed regimens or details regarding exact timing for initiation of treatment. As this study is retrospective in nature, it is also unable to determine causal relationships, only correlations. Lastly, while propensity matching our cohorts attempts to limit any confounders, unexamined variables in addition to BP usage may have influenced results.

## Conclusion

BP-managed OP was associated with lower rates of periprosthetic fracture, osteolysis, and revision TSA within 90 days, revision TSA at 1 year, and PJI and mechanical loosening at both 1 and 2 years. These results suggest that perioperative BP therapy for osteoporotic patients undergoing shoulder arthroplasty, when medically indicated, may be beneficial in reducing postoperative complications. Future studies should evaluate TSA outcomes in osteoporotic patients medically managed with different classes of OP medications like parathyroid hormone analogs or monoclonal antibodies. Furthermore, prospective investigations could ascertain the direct effects of preoperative vs. postoperative initiation of BPs on TSA outcomes.

## Disclaimers:

Funding: No funding was disclosed by the authors.

Conflicts of interest: The authors, their immediate families, and any research foundations with which they are affiliated have not received any financial payments or other benefits from any commercial entity related to the subject of this article.
