# Supplementary material for: Shoulder arthroplasty in the osteoporotic patient: do bisphosphonates make a difference?
Source: JSES Int. 2026 Jan 28;10(3):101629. doi: 10.1016/j.jseint.2026.101629 (PMC12993121; doi:10.1016/j.jseint.2026.101629)
Supplement: Supplemental File [file mmc1.docx]

Supplementary Material

| **Supplementary Figure 1.** Propensity score density function before and after matching (BP group = purple; noTx group = green), demonstrating improved overlap after adjustment for covariates listed in Supplementary Tables 1 and 2 |
| --- |


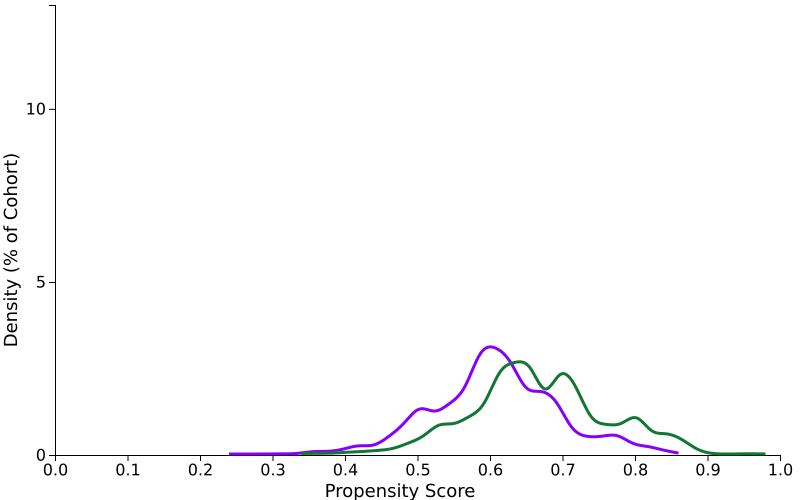

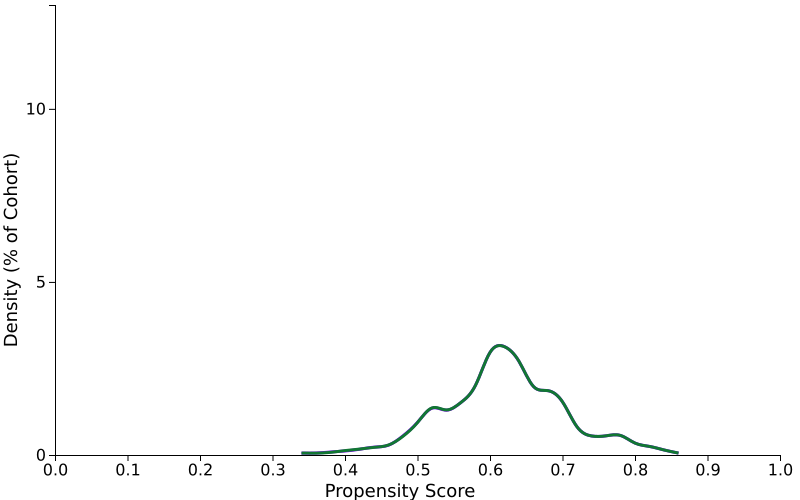


After

Before

| **Supplementary Table 1.** BP group (Cohort 1, N = 3,642) and noTx group (Cohort 2, N = 6,775) characteristics before propensity score matching | | | | | | | | | |
| --- | --- | --- | --- | --- | --- | --- | --- | --- | --- |
|  | **Demographics** | | | | | | | | |
|  |  | Cohort | |  | Mean ± SD | Patients | % of Cohort | P-Value | Std diff. |
|  |  | 1 2 | AI | Age at Index | 73.6 +/- 8.6 73.3 +/- 9.4 | 3,628 6,750 | 100% 100% | 0.074 | 0.037 |
|  |  | 1 2 | 2106-3 | White |  | 3,053 5,457 | 84.2% 80.8% | <0.001 | 0.087 |
|  |  | 1 2 | UNK | Unknown Race |  | 228 511 | 6.3% 7.6% | 0.015 | 0.051 |
|  |  | 1 2 | F | Female |  | 3,138 5,149 | 86.5% 76.3% | <0.001 | 0.265 |
|  |  | 1 2 | 2054-5 | Black or African American |  | 203 396 | 5.6% 5.9% | 0.572 | 0.012 |
|  |  | 1 2 | M | Male |  | 346 1,167 | 9.5% 17.3% | <0.001 | 0.229 |
|  |  | 1 2 | 2028-9 | Asian |  | 44 91 | 1.2% 1.3% | 0.562 | 0.012 |
|  | **Diagnosis** | | | | | | | | |
|  |  | Cohort | |  | Mean ± SD | Patients | % of Cohort | P-Value | Std diff. |
|  |  | 1 2 | Z72.0 | Tobacco use |  | 95 144 | 2.6% 2.1% | 0.116 | 0.032 |
|  |  | 1 2 | I21 | Acute myocardial infarction |  | 122 198 | 3.4% 2.9% | 0.228 | 0.025 |
|  |  | 1 2 | I50.9 | Heart failure, unspecified |  | 273 538 | 7.5% 8.0% | 0.420 | 0.017 |
|  |  | 1 2 | I73.9 | Peripheral vascular disease, unspecified |  | 205 335 | 5.7% 5.0% | 0.133 | 0.031 |
|  |  | 1 2 | I67 | Other cerebrovascular diseases |  | 139 225 | 3.8% 3.3% | 0.189 | 0.027 |
|  |  | 1 2 | F03 | Unspecified dementia |  | 100 178 | 2.8% 2.6% | 0.720 | 0.007 |
|  |  | 1 2 | J44 | Other chronic obstructive pulmonary disease |  | 579 944 | 16.0% 14.0% | 0.007 | 0.055 |
|  |  | 1 2 | M35 | Other systemic involvement of connective tissue |  | 211 240 | 5.8% 3.6% | <0.001 | 0.107 |
|  |  | 1 2 | K27 | Peptic ulcer, site unspecified |  | 44 54 | 1.2% 0.8% | 0.038 | 0.041 |
|  |  | 1 2 | K76 | Other diseases of liver |  | 218 353 | 6.0% 5.2% | 0.097 | 0.034 |
|  |  | 1 2 | E11 | Type 2 diabetes mellitus |  | 792 1,529 | 21.8% 22.7% | 0.338 | 0.020 |
|  |  | 1 2 | E10 | Type 1 diabetes mellitus |  | 64 111 | 1.8% 1.6% | 0.652 | 0.009 |
|  |  | 1 2 | G81 | Hemiplegia and hemiparesis |  | 16 19 | 0.4% 0.3% | 0.181 | 0.027 |
|  |  | 1 2 | N18 | Chronic kidney disease (CKD) |  | 554 1,058 | 15.3% 15.7% | 0.588 | 0.011 |
|  |  | 1 2 | C80 | Malignant neoplasm without specification of site |  | 42 50 | 1.2% 0.7% | 0.031 | 0.043 |
|  |  | 1 2 | C95 | Leukemia of unspecified cell type |  | 10 10 | 0.3% 0.1% | 0.158 | 0.028 |
|  |  | 1 2 | C85 | Other specified and unspecified types of non-Hodgkin lymphoma |  | 23 47 | 0.6% 0.7% | 0.711 | 0.008 |
|  |  | 1 2 | C81 | Hodgkin lymphoma |  | 0 10 | 0% 0.1% | 0.020 | 0.054 |
|  |  | 1 2 | B20-B20 | Human immunodeficiency virus [HIV] disease (B20) |  | 10 19 | 0.3% 0.3% | 0.957 | 0.001 |
|  | **Laboratory** | | | | | | | | |
|  |  | Cohort | |  | Mean ± SD | Patients | % of Cohort | P-Value | Std diff. |
|  |  | 1 2 | 9083 | BMI | 28.5 +/- 6.7 29.2 +/- 7.0 | 2,979 4,956 | 82.1% 73.4% | <0.001 | 0.109 |
|  |  | 1 2 |  | 0 - 18.50 kg/m2 |  | 153 225 | 4.2% 3.3% | 0.022 | 0.046 |
|  |  | 1 2 |  | 18.50 - 25 kg/m2 |  | 1,166 1,696 | 32.1% 25.1% | <0.001 | 0.156 |
|  |  | 1 2 |  | 25 - 30 kg/m2 |  | 1,299 2,017 | 35.8% 29.9% | <0.001 | 0.126 |
|  |  | 1 2 |  | 30 - 0 kg/m2 |  | 1,297 2,335 | 35.7% 34.6% | 0.239 | 0.024 |

| **Supplementary Table 2.** BP group (Cohort 1, N = 3,604) and noTx group (Cohort 2, N = 3,604) characteristics after propensity score matching | | | | | | | | | |
| --- | --- | --- | --- | --- | --- | --- | --- | --- | --- |
|  | **Demographics** | | | | | | | | |
|  |  | Cohort | |  | Mean ± SD | Patients | % of Cohort | P-Value | Std diff. |
|  |  | 1 2 | AI | Age at Index | 73.6 +/- 8.6 73.6 +/- 9.0 | 3,604 3,604 | 100% 100% | 0.877 | 0.004 |
|  |  | 1 2 | 2106-3 | White |  | 3,030 3,061 | 84.1% 84.9% | 0.313 | 0.024 |
|  |  | 1 2 | UNK | Unknown Race |  | 227 229 | 6.3% 6.4% | 0.923 | 0.002 |
|  |  | 1 2 | F | Female |  | 3,114 3,127 | 86.4% 86.8% | 0.653 | 0.011 |
|  |  | 1 2 | 2054-5 | Black or African American |  | 203 196 | 5.6% 5.4% | 0.718 | 0.008 |
|  |  | 1 2 | M | Male |  | 346 346 | 9.6% 9.6% | 1 | <0.001 |
|  |  | 1 2 | 2028-9 | Asian |  | 44 30 | 1.2% 0.8% | 0.102 | 0.039 |
|  | **Diagnosis** | | | | | | | | |
|  |  | Cohort | |  | Mean ± SD | Patients | % of Cohort | P-Value | Std diff. |
|  |  | 1 2 | Z72.0 | Tobacco use |  | 93 84 | 2.6% 2.3% | 0.493 | 0.016 |
|  |  | 1 2 | I21 | Acute myocardial infarction |  | 121 111 | 3.4% 3.1% | 0.505 | 0.016 |
|  |  | 1 2 | I50.9 | Heart failure, unspecified |  | 272 254 | 7.5% 7.0% | 0.415 | 0.019 |
|  |  | 1 2 | I73.9 | Peripheral vascular disease, unspecified |  | 200 169 | 5.5% 4.7% | 0.098 | 0.039 |
|  |  | 1 2 | I67 | Other cerebrovascular diseases |  | 138 116 | 3.8% 3.2% | 0.160 | 0.033 |
|  |  | 1 2 | F03 | Unspecified dementia |  | 99 81 | 2.7% 2.2% | 0.174 | 0.032 |
|  |  | 1 2 | J44 | Other chronic obstructive pulmonary disease |  | 569 547 | 15.8% 15.2% | 0.474 | 0.017 |
|  |  | 1 2 | M35 | Other systemic involvement of connective tissue |  | 202 176 | 5.6% 4.9% | 0.170 | 0.032 |
|  |  | 1 2 | K27 | Peptic ulcer, site unspecified |  | 41 35 | 1.1% 1.0% | 0.489 | 0.016 |
|  |  | 1 2 | K76 | Other diseases of liver |  | 213 208 | 5.9% 5.8% | 0.802 | 0.006 |
|  |  | 1 2 | E11 | Type 2 diabetes mellitus |  | 783 782 | 21.7% 21.7% | 0.977 | 0.001 |
|  |  | 1 2 | E10 | Type 1 diabetes mellitus |  | 64 50 | 1.8% 1.4% | 0.186 | 0.031 |
|  |  | 1 2 | G81 | Hemiplegia and hemiparesis |  | 15 15 | 0.4% 0.4% | 1 | <0.001 |
|  |  | 1 2 | N18 | Chronic kidney disease (CKD) |  | 550 544 | 15.3% 15.1% | 0.844 | 0.005 |
|  |  | 1 2 | C80 | Malignant neoplasm without specification of site |  | 36 34 | 1.0% 0.9% | 0.810 | 0.006 |
|  |  | 1 2 | C95 | Leukemia of unspecified cell type |  | 10 10 | 0.3% 0.3% | 1 | <0.001 |
|  |  | 1 2 | C85 | Other specified and unspecified types of non-Hodgkin lymphoma |  | 23 18 | 0.6% 0.5% | 0.434 | 0.018 |
|  |  | 1 2 | C81 | Hodgkin lymphoma |  | 0 0 | 0% 0% | -- | -- |
|  |  | 1 2 | B20-B20 | Human immunodeficiency virus [HIV] disease (B20) |  | 10 10 | 0.3% 0.3% | 1 | <0.001 |
|  | **Laboratory** | | | | | | | | |
|  |  | Cohort | |  | Mean ± SD | Patients | % of Cohort | P-Value | Std diff. |
|  |  | 1 2 | 9083 | BMI | 28.5 +/- 6.7 28.7 +/- 6.8 | 2,955 2,961 | 82.0% 82.2% | 0.435 | 0.020 |
|  |  | 1 2 |  | 0 - 18.50 kg/m2 |  | 151 146 | 4.2% 4.1% | 0.767 | 0.007 |
|  |  | 1 2 |  | 18.50 - 25 kg/m2 |  | 1,148 1,161 | 31.9% 32.2% | 0.743 | 0.008 |
|  |  | 1 2 |  | 25 - 30 kg/m2 |  | 1,278 1,305 | 35.5% 36.2% | 0.507 | 0.016 |
|  |  | 1 2 |  | 30 - 0 kg/m2 |  | 1,284 1,285 | 35.6% 35.7% | 0.980 | 0.001 |

| **Supplementary Table 3.** Text representation of the cohort definitions | | | | |
| --- | --- | --- | --- | --- |
| **Cohort** | **Patients cannot have any of the following:** | **All the following must be satisfied:** | |  |
| Bisphosphonate-treated group (BP group) | Injection, teriparatide, 10 mcg (UMLS:HCPCS:J3110); or teriparatide (NLM:RXNORM:32915); or Injection, romosozumab-aqqg, 1 mg (UMLS:HCPCS:J3111); or abaloparatide (NLM:RXNORM:1921069); or romosozumab (NLM:RXNORM:2123126); or Arthroplasty, glenohumeral joint; hemiarthroplasty (UMLS:CPT:23470). | **TSA:** The terms in this group occurred at any time. Patients must have any of the following: Arthroplasty, glenohumeral joint; total shoulder (glenoid and proximal humeral replacement (eg, total shoulder)) (UMLS:CPT:23472); or Presence of artificial shoulder joint (UMLS:ICD10CM:Z96.61).  **Bisphos:** Any instance of Bisphos occurred within 3 years before or up to 3 years after any instance of TSA. Patients must have any of the following: Injection, ibandronate sodium, 1 mg (UMLS:HCPCS:J1740); or zoledronic acid (NLM:RXNORM:77655); or ibandronate (NLM:RXNORM:115264); or Injection, zoledronic acid, 1 mg (UMLS:HCPCS:J3489); or pamidronate (NLM:RXNORM:11473); or etidronate (NLM:RXNORM:42682); or risedronate (NLM:RXNORM:73056); or alendronate (NLM:RXNORM:46041).  **OP:** Any instance of OP occurred within 3 months on or before any instance of TSA. Patients must have any of the following: Osteoporosis with current pathological fracture (UMLS:ICD10CM:M80); or Osteoporosis without current pathological fracture (UMLS:ICD10CM:M81).  **2yr fu:** Any instance of 2yr fu occurred at least 2 years after any instance of TSA. Patients must have: Visit (TNX:Visit). |  |  |
| No treatment group (noTx group) | teriparatide (NLM:RXNORM:32915); or Injection, teriparatide, 10 mcg (UMLS:HCPCS:J3110); or pamidronate (NLM:RXNORM:11473); or Injection, romosozumab-aqqg, 1 mg (UMLS:HCPCS:J3111); or etidronate (NLM:RXNORM:42682); or alendronate (NLM:RXNORM:46041); or risedronate (NLM:RXNORM:73056); or romosozumab (NLM:RXNORM:2123126); or zoledronic acid (NLM:RXNORM:77655); or Injection, zoledronic acid, 1 mg (UMLS:HCPCS:J3489); or abaloparatide (NLM:RXNORM:1921069); or Injection, ibandronate sodium, 1 mg (UMLS:HCPCS:J1740); or ibandronate (NLM:RXNORM:115264); or Arthroplasty, glenohumeral joint; hemiarthroplasty (UMLS:CPT:23470). | **TSA:** The terms in this group occurred at any time. Patients must have any of the following: Arthroplasty, glenohumeral joint; total shoulder (glenoid and proximal humeral replacement (eg, total shoulder)) (UMLS:CPT:23472); or Presence of artificial shoulder joint (UMLS:ICD10CM:Z96.61).  **OP:** Any instance of OP occurred within 3 months on or before any instance of TSA. Patients must have any of the following: Osteoporosis without current pathological fracture (UMLS:ICD10CM:M81); or Osteoporosis with current pathological fracture (UMLS:ICD10CM:M80).  **2yr fu:** Any instance of 2yr fu occurred at least 2 years after any instance of TSA. Patients must have: Visit (TNX:Visit). |  |  |

| **Supplementary Table 4.** Text representation of the outcome definitions | | | |
| --- | --- | --- | --- |
| **Outcome** | **Patients must have any of the following:** | |  |
| Dislocation | Dislocation of other internal joint prosthesis (UMLS:ICD10CM:T84.028); or Dislocation of unspecified internal joint prosthesis (UMLS:ICD10CM:T84.029). |  |  |
| Intraoperative fracture | Fracture of humerus following insertion of orthopedic implant, joint prosthesis, or bone plate (UMLS:ICD10CM:M96.62). |  |  |
| Mechanical loosening | Mechanical loosening of other internal prosthetic joint (UMLS:ICD10CM:T84.038); or Mechanical loosening of unspecified internal prosthetic joint (UMLS:ICD10CM:T84.039). |  |  |
| Osteolysis | Osteolysis, shoulder (UMLS:ICD10CM:M89.51); or Periprosthetic osteolysis of other internal prosthetic joint (UMLS:ICD10CM:T84.058); or Periprosthetic osteolysis of unspecified internal prosthetic joint (UMLS:ICD10CM:T84.059). |  |  |
| Periprosthetic fracture | Periprosthetic fracture around internal prosthetic shoulder joint (UMLS:ICD10CM:M97.3); or Fracture of humerus following insertion of orthopedic implant, joint prosthesis, or bone plate (UMLS:ICD10CM:M96.62). |  |  |
| Postoperative infection | Infection following a procedure (UMLS:ICD10CM:T81.4). |  |  |
| Prosthetic joint infection (PJI) | Infection and inflammatory reaction due to unspecified internal joint prosthesis (UMLS:ICD10CM:T84.50); or Infection and inflammatory reaction due to other internal joint prosthesis (UMLS:ICD10CM:T84.59). |  |  |
| Revision surgery | Revision of total shoulder arthroplasty, including allograft when performed; humeral or glenoid component (UMLS:CPT:23473); or Revision of total shoulder arthroplasty, including allograft when performed; humeral and glenoid component (UMLS:CPT:23474); or Removal of prosthesis, includes debridement and synovectomy when performed; humeral or glenoid component (UMLS:CPT:23334); or Removal of prosthesis, includes debridement and synovectomy when performed; humeral and glenoid components (eg, total shoulder) (UMLS:CPT:23335). |  |  |
